# Chemerin: a multifaceted adipokine involved in metabolic disorders

**DOI:** 10.1530/JOE-18-0174

**Published:** 2018-05-30

**Authors:** Gisela Helfer, Qing-Feng Wu

**Affiliations:** 1 School of Chemistry and BiosciencesUniversity of Bradford, Bradford, UK; 2 State Key Laboratory of Molecular Development BiologyInstitute of Genetics and Developmental Biology, Chinese Academy of Sciences, Beijing, China

**Keywords:** chemerin, CMKLR1, GPR1, energy balance, glucose homeostasis, whole body metabolism, hypothalamus, tanycytes

## Abstract

Metabolic syndrome is a global public health problem and predisposes individuals to obesity, diabetes and cardiovascular disease. Although the underlying mechanisms remain to be elucidated, accumulating evidence has uncovered a critical role of adipokines. Chemerin, encoded by the gene *Rarres2*, is a newly discovered adipokine involved in inflammation, adipogenesis, angiogenesis and energy metabolism. In humans, local and circulating levels of chemerin are positively correlated with BMI and obesity-related biomarkers. In this review, we discuss both peripheral and central roles of chemerin in regulating body metabolism. In general, chemerin is upregulated in obese and diabetic animals. Previous studies by gain or loss of function show an association of chemerin with adipogenesis, glucose homeostasis, food intake and body weight. In the brain, the hypothalamus integrates peripheral afferent signals including adipokines to regulate appetite and energy homeostasis. Chemerin increases food intake in seasonal animals by acting on hypothalamic stem cells, the tanycytes. In peripheral tissues, chemerin increases cell expansion, inflammation and angiogenesis in adipose tissue, collectively resulting in adiposity. While chemerin signalling enhances insulin secretion from pancreatic islets, contradictory results have been reported on how chemerin links to obesity and insulin resistance. Given the association of chemerin with obesity comorbidities in humans, advances in translational research targeting chemerin are expected to mitigate metabolic disorders. Together, the exciting findings gathered in the last decade clearly indicate a crucial multifaceted role for chemerin in the regulation of energy balance, making it a promising candidate for urgently needed pharmacological treatment strategies for obesity.

## Introduction

**Figure 1:**
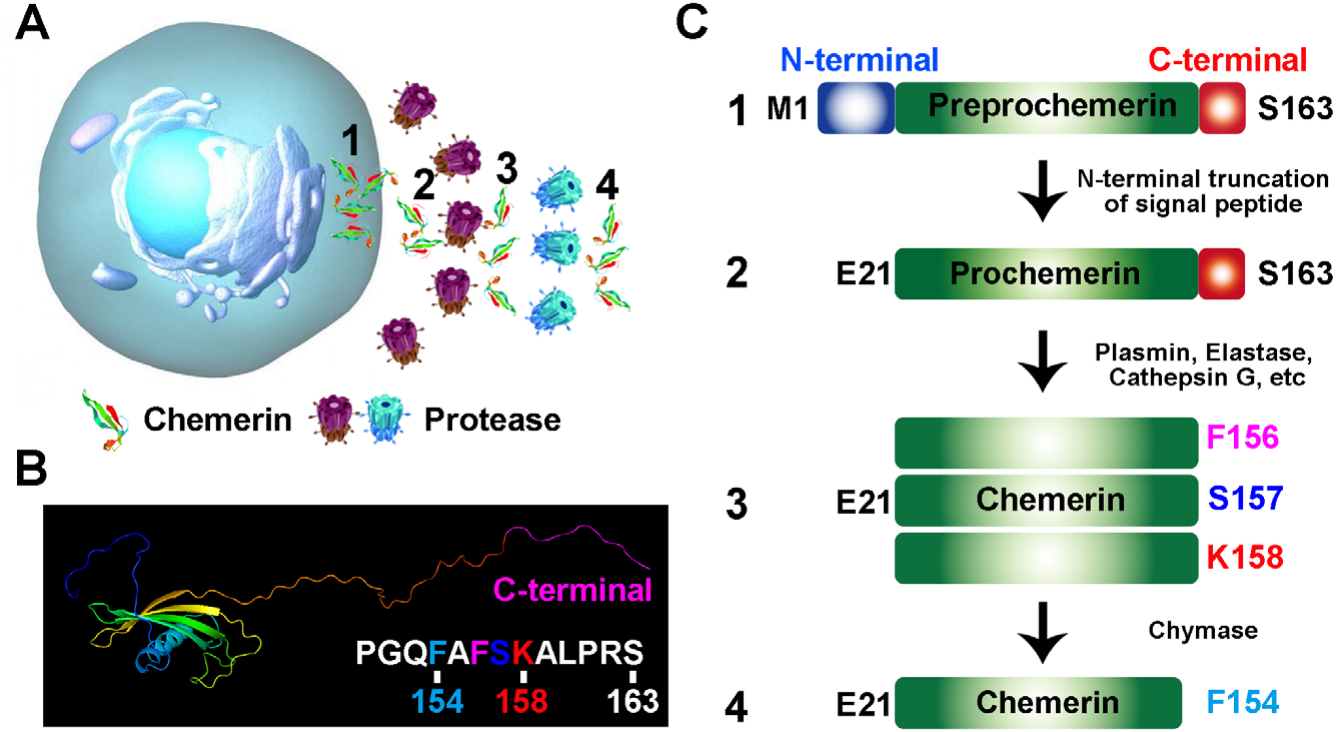
Processing of prochemerin. (A) The scheme describes the release of preprochemerin (1), proteolysis of prochemerin (2) into bioactive chemerin (3) and transformation of active isoform into deactivated chemerin (4). (B) The predicted 3D structure of chemerin from RaptorX structure prediction server. (C) The processing of prochemerin by various proteases.

Adipokines, secreted by adipose tissue, are involved in the pathogenesis of metabolic syndrome (Lehr* et al*. 2012). Chemerin, encoded by the gene retinoic acid receptor responder 2 (*Rarres2*), also known as tazarotene-induced gene 2 (TIG2), was recently identified as one of the adipokines with autocrine, paracrine and even endocrine roles *in vivo* (Rourke* et al*. 2013). Chemerin is an inflammatory chemokine and was originally discovered as a novel retinoic acid-responsive gene in psoriatic skin lesions, implying an immunomodulating role (Nagpal* et al*. 1997). Initially, chemerin was termed TIG2 because an increased expression was reported after treatment of skin raft cultures with tazarotone, a synthetic retinoid (Nagpal* et al*. 1997). It was further characterised as a retinoid-responsive gene and thereby called retinoic acid receptor responder 2 (*Rarres2*). Retinoic acid responsiveness of chemerin was later confirmed in various tissues and cells (Nagpal* et al*. 1997, Martensson* et al*. 2005, Gonzalvo-Feo* et al*. 2014, Helfer* et al*. 2016). Further studies revealed that chemerin was highly expressed in white adipose tissue (WAT), liver and lung while its receptor CMKLR1 is predominantly expressed in adipocyte and immune cells (Bozaoglu* et al*. 2007, Goralski* et al*. 2007). In mammalian cells, chemerin is initially synthesised as a 163 amino acid (aa) proprecursor. The N-terminal truncation of 20 aa signal peptide results in the release of inactive precursor (chemerin-S163) into extracellular niches or circulation system (Fig. 1A). The precursor requires further extracellular C-terminal cleavage at various sites to generate active and deactivated chemerin. For example, the proteolytic cleavage at its C-terminus by plasmin, elastase and cathepsin G activates chemerin and generates various isoforms (chemerin-K158, -S157 and -F156) with different affinity to CMKLR1 (Fig. 1B and C). Further cleavage of bioactive chemerin by chymase produces chemerin-F154 and terminates its activity (Mattern* et al*. 2014). Thus, the C-terminal proteolytic processing serves as a key regulatory mechanism to determine the local and systemic concentration of active chemerin. To date, eight serine proteases have been identified to C-terminally process chemerin and its precursor *in vitro*. These serine proteases are generally secreted into extracellular matrix or blood plasma to exert their biological effect (Mattern* et al*. 2014).

After secretion, chemerin precursor is processed by various extracellular proteases of the coagulation, fibrinolytic and inflammatory cascades, which are differentially expressed in a wide range of tissues and thereby produce distinct chemerin isoforms. Although inert chemerin precursor is the dominant isoform in plasma from healthy humans, differential chemerin isoforms in human blood (chemerin-A155, -S157 and -K158), cerebrospinal fluid (chemerin-K158), ascites (chemerin-S157), hemofiltrate (chemerin-F154) and synovial fluid (chemerin-K158) under inflammatory conditions have been detected (Rourke* et al*. 2014). Results from mass spectrometry analysis indicate that bioactive chemerin generation takes place at early stages of inflammation (Wittamer* et al*. 2005). ELISAs using antibodies raised against C-terminal peptides have shown that Chemerin-K158 is the dominant isoform in the synovial fluid of patients with arthritis (Zhao* et al*. 2011). Further analysis reveals that the fraction of bioactive chemerin is much higher in the inflammatory cerebrospinal fluid and synovial fluid from patients than the plasma from healthy volunteers (Zhao* et al*. 2011). In the adipose tissue of patients with obesity, chemerin isoforms display a different profile from plasma, with minimal chemerin precursor and significant levels of bioactive chemerin-S157 (Chang* et al*. 2016). These results suggest that complex chemerin processing occurs *in vivo*, especially during inflammation. Mass spectrometry analysis is required to determine the profile of chemerin isoforms in other parenchymal tissues such as liver and lung.

Chemerin has been shown to display various roles in the pathogenesis of inflammatory and metabolic disease in multiple organs such as adipose tissue, lung, skin, cardiovascular system, reproductive tract, digestive tract, skeleton and joints. The biological function of chemerin as pro- or anti-inflammatory modulators remains controversial. At the onset of inflammatory reaction, polymorphonuclear cells are firstly recruited to the damaged sites, where they promote the generation of bioactive chemerin by releasing proteases including elastase and cathepsin G into the milieu (Wittamer* et al*. 2003, 2005). Subsequently, chemerin functions to enhance the chemotaxis of immature dendritic cells and macrophages, bridging innate and adaptive immunity for the initiation of immune response. In contrast, chemerin treatment reduces the recruitment of neutrophil and macrophages to the inflammatory sites and the expression of proinflammatory cytokine (Cash* et al*. 2008, Luangsay* et al*. 2009). These studies suggest that chemerin acts as either a proinflammatory or anti-inflammatory modulator depending on the biological systems. A fundamental switch in our understanding of chemerin’s function occurred in 2007 when chemerin was found to be highly expressed in WAT (Goralski* et al*. 2007). Subsequent studies revealed that chemerin acts on its receptor CMKLR1 to affect adipogenesis, angiogenesis and inflammation in adipose tissue. Beyond the lipid metabolism, chemerin also influences the dysregulation of glucose metabolism. Supporting the important roles of chemerin in systemic lipid and glucose metabolism, accumulating clinical data indicate that local and/or circulating chemerin levels are increased in patients with obesity, diabetes and cardiovascular disease (Perumalsamy* et al*. 2017). Given the scope of this review, we are focusing exclusively on the role of chemerin in regulating metabolism.

## Chemerin and its receptors

As a chemoattractant protein, chemerin was first identified as the natural ligand for the G-protein-coupled receptor CMKLR1, also known as ChemR23 (Meder* et al*. 2003, Wittamer* et al*. 2003). Later, owing to a high sequence identity, chemerin was also recognised as the ligand for another G protein-coupled receptor GPR1 (Barnea* et al*. 2008, Southern* et al*. 2013, Rourke* et al*. 2014). The third chemerin receptor chemokine (C-C motif) receptor-like 2 (Ccrl2) was identified in experiments based on binding assays (Zabel* et al*. 2008). Recently, De Henau* et al*. generated cell lines expressing each individual chemerin receptor and compared their binding and signalling properties separately of cellular context. Chemerin binds to both CMKLR1 and GPR1 with similar affinity, but lower affinity to CCRL2 (De Henau* et al*. 2016).

Chemerin receptor signalling has been reviewed in detail recently (Mattern* et al*. 2014, Kennedy & Davenport 2018) and therefore we only provide a short summary here before we discuss the role of chemerin and its receptors in energy balance regulation. Chemerin receptors display cell-specific expression profiles, thus making a comparison of receptor activation in the same tissue difficult (Kennedy & Davenport 2018). CMKLR1 expression is wide-spread in different organs and tissues. It is expressed in the adaptive immune system, with high level of transcripts detected in macrophages, natural killer cells, immature dendritic cells and leucocytes (Samson* et al*. 1998, Wittamer* et al*. 2003, Vermi* et al*. 2005, Parolini* et al*. 2007). Additionally, it has been detected in the cardiovascular system (including smooth muscle cells, endothelial cells and cardiomyocytes), the reproductive system (such as Leydig cells) and the skin (Li* et al*. 2014, Banas* et al*. 2015, Kennedy* et al*. 2016). In line with chemerin’s role as an adipokine, CMKLR1 is expressed in adipocytes, with higher levels in white compared to brown adipose tissue (BAT) (Goralski* et al*. 2007). In the brain, CMKLR1 is found in microglia of the hippocampus as well as ependymal cells and tanycytes lining the third ventricle of the hypothalamus (Guo* et al*. 2012, Helfer* et al*. 2016). CMKLR1 is a G_i/o_-protein-coupled receptor that signals through mitogen-activated protein kinase (MAPK), extracellular signal-regulated kinases (ERK) and phosphatidylinositol 3 kinase (PI3K)-AKT pathways to regulate biological functions such as angiogenesis and inflammation (Wittamer* et al*. 2003, Goralski* et al*. 2007, Sell* et al*. 2009, De Henau* et al*. 2016).

GPR1 is predominantly expressed in central nervous system, such as glioblastoma cells, brain-derived fibroblast-like cells lines and microglia although expression was also reported in skin cells, white adipocytes, Leydig cells and granulosa cells (Edinger* et al*. 1998, Shimizu* et al*. 1999, Reverchon* et al*. 2012, Li* et al*. 2014, Banas* et al*. 2015). Limited studies have investigated signalling transduction properties of GPR1 upon activation of chemerin. As with CMKLR1, GPR1 activates ERK1/2-MAPK pathways (Rourke* et al*. 2015, De Henau* et al*. 2016). Furthermore, binding of chemerin to CMKLR1 and GPR1 promotes RhoA/ROCK-dependent pathways (Rourke* et al*. 2015).

Unlike CMKLR1 and GPR1, the binding of chemerin to CCRL2 does not induce downstream signalling pathways, calcium mobilisation or ligand internalisation, and it is therefore designated as an atypical, silent or non-signalling chemokine receptor. CCRL2 seems to have the ability to amplify local chemerin concentration for CMKLR1 interaction (Zabel* et al*. 2008, De Henau* et al*. 2016).

## The role of chemerin in energy balance regulation and obesity

### Effect on whole body metabolism

After the discovery of leptin, adipokines have been increasingly found to have many effects on biological functions including blood pressure, homeostasis, adipogenesis and glucose metabolism linking adipokines to the metabolic syndrome. Several studies have suggested that the adipokine chemerin plays a crucial role in adipogenesis, and this has been implicated in the control of adipose tissue in regard to the regulation of glucose homeostasis and the development of obesity.

In general, contradictory results have been reported on what role chemerin plays in whole body metabolism and how it links to obesity and insulin resistance. Initially, studies in humans indicated that chemerin gene expression and circulating levels are positively correlated with increased BMI and obesity-related biomarkers (Bozaoglu* et al*. 2007, 2009, Sell* et al*. 2009, Chakaroun* et al*. 2012). In support, plasma chemerin levels are increased in diet-induced obese mice and reduced by overnight fasting. This effect is independent of the strain of mice (FVB and C57BL/6) used (Wargent* et al*. 2015). In contrast, there was no change of plasma chemerin levels in NMRI mice on a high-fat or cafeteria diet (Hansen* et al*. 2014). This discrepancy might be explained by different strains of mice being more susceptible to diet-induced obesity than others. Plasma chemerin levels were raised in genetically obese (ob/ob) mice (Ernst* et al*. 2010), whereas leptin receptor-deficient mice (db/db mice) had decreased levels of serum chemerin concentration and displayed an increase in insulin signalling (Takahashi* et al*. 2008). In rats maintained on a restricted diet, a decrease in the level of chemerin (*Rarres2*) mRNA in the WAT was associated with a decrease of serum chemerin concentration. When these rats were re-fed after diet restriction, both chemerin expression in WAT and serum chemerin concentration were upregulated (Stelmanska* et al*. 2013). Surprisingly, intraperitoneal injections of chemerin into rats resulted in a lower body weight (Brunetti* et al*. 2014). This seems to contradict the notion that raised plasma chemerin levels promote obesity. Our study using chemerin injections directly into the brain suggested that there is a bimodal response on body weight and food intake. Acute intracerebroventricular bolus injection of chemerin decreased body weight while chronic chemerin infusion increased body weight (Helfer* et al*. 2016). Thus, chemerin might exert different biological actions depending on the timeframe investigated.

Given that most studies report increased chemerin levels with increased body weight, a long-term proinflammatory effect might link it to insulin resistance in obesity. While intraperitoneal injections of chemerin into normal mice had no effect on glucose tolerance, there was increased glucose intolerance in ob/ob mice and db/db mice (Ernst* et al*. 2010). Further indications linking chemerin with glucose homeostasis were provided by studies using mice lacking the GPR1 receptor. *Gpr1*-knockout mice on a high-fat diet showed increased glucose intolerance compared to wild-type mice, but displayed no change in body weight, body composition and energy expenditure (Rourke* et al*. 2014) (explained in detail later).

Similarly, studies using *Cmkrl1*-knockout mice have reported inconsistent metabolic phenotypes. *Cmklr1*-knockout mice have reduced food intake and body weight compared to wild-type mice when fed on either a low- or a high-fat diet (Ernst* et al*. 2012) but other studies have found no or little effect on body composition and glucose homeostasis (Rouger* et al*. 2013, Gruben* et al*. 2014). The differences reported might have been due to age and sex of the mice and different lengths and diets used in these studies. Recently, Wargent* et al*. have attempted to clarify the situation and found that male and female *Cmklr1*-knockout mice on a high-fat diet have mildly increased body fat and impaired glucose homeostasis, although this is age dependent (Wargent* et al*. 2015). Thus, it is unclear whether raised chemerin levels promote obesity and more studies are needed to provide a definite answer.

### Effect on the brain

**Figure 2:**
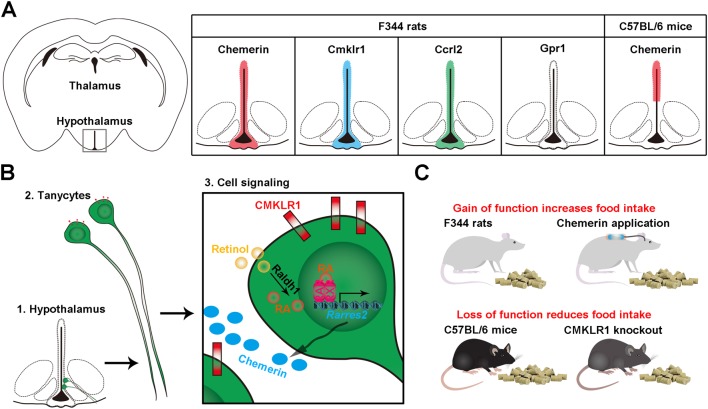
The expression, signalling and function of chemerin in the hypothalamus. (A) Chemerin, *Cmklr1*, *Ccrl2* but not *Gpr1* are expressed in the tanycytes and ependymal cells lining the third ventricle of the hypothalamus of F344 rats (Helfer* et al*. 2016). In C57BL/6 mice, chemerin expression is restricted to ependymal cells (Miranda-Angulo* et al*. 2014). (B) In tanycytes, chemerin is downstream of retinoic acid signalling. Retinol enters the tanycytes where it is synthesised to retinoic acid (RA). RA enters the nucleus and binding to its receptors RAR and RXR leads to transcription of *Rarres2*, which is translated into* c*hemerin. Chemerin binds to its receptor CMKLR1 and activates its downstream signalling pathway in an autocrine or paracrine manner. (C) In general, long-term application of chemerin increases food intake and loss of function reduces food intake, although contradictory results have been reported.

Chemerin and its receptors are localised in distinct brain regions indicating a potential central role. In mice and rats, chemerin (*Rarres2*) mRNA is expressed in the hypothalamus (Miranda-Angulo* et al*. 2014, Helfer* et al*. 2016). In rats, *Cmklr1* transcript was found in the prefrontal cortex, hippocampus, cerebellum and hypothalamus (Guo* et al*. 2012, Helfer* et al*. 2016). Both CMKLR1 and GPR1 are highly expressed in the mouse brain, but the tissue specificity has not been identified among different brain regions (Rourke* et al*. 2014). Although it has been reported that hypothalamic nuclei express *Gpr1* in particularly high levels, *Gpr1* mRNA were not found to be expressed in the rat hypothalamus using *in situ* hybridisation (Helfer* et al*. 2016). In GPR1 knockout mice, chemerin (*Rarres2*) mRNA expression is increased in the hypothalamus, whereas CMKLR1 expression is simultaneously decreased in the cortex and hypothalamus (Fig. 2A) (Rourke* et al*. 2014).

Given the important role of chemerin in energy balance regulation and obesity, surprisingly little is known about its expression and function in the hypothalamus, the key area of energy balance regulation. In the brain, the hypothalamus is critical in sensing and integrating peripheral signals such as adipokines (Coll & Yeo 2013). In the hypothalamus of rats, chemerin and *Cmklr1* (but not *Gpr1*) transcripts were localised in the ependymal cells and tanycytes, specialised glial cells lining the third ventricle and extending into the arcuate nucleus, hypothalamic areas closely associated with homeostatic appetite regulation. The third chemerin receptor CCRL2 was also found in the same hypothalamic loci, possibly increasing the local chemerin signal (Helfer* et al*. 2016). In contrast, in mice, chemerin is predominantly expressed in ependymal cells but not in the tanycytes (Miranda-Angulo* et al*. 2014). The observation that chemerin is not expressed uniformly in the hypothalamus might suggest that it plays different roles in different locations (Fig. 2A); however, a potential species-specific expression pattern needs to be confirmed in further studies.

An initial link between chemerin and appetite and body weight regulation in the hypothalamus was identified in genome-wide expression analysis (Ross* et al*. 2011). Further investigations identified chemerin as downstream target of retinoic acid signalling in the hypothalamus in photoperiod-sensitive F344 rats (Fig. 2B). Intracerebroventricular injections of all-trans retionic acid into the third ventricle of F344 rats increased chemerin mRNA expression in ependymal cells and tanycytes lining the third ventricle (Helfer* et al*. 2016). Photoperiod-sensitive mammals can be stimulated to make marked physiological changes in body weight and food intake status simply by changing photoperiod (daylength); thus, they are used as a natural model of obesity (Ebling 2014). While laboratory rats are generally not responsive to photoperiod, the F344 rat is one of the few rat strains that retained its photoperiod sensitivity and undergoes pronounced cycles of weight gain and weight loss under different photoperiod conditions (Tavolaro* et al*. 2015). In the hypothalamus of F344 rats, chemerin is strongly regulated by photoperiodic changes. Furthermore, intracerebroventricular administration of chemerin into F344 rats alters food intake and body weight with associated changes in hypothalamic neuropeptides involved in feeding and growth (Fig. 2C). However, in F344 rats, this response is transient and not sustained over long term (Helfer* et al*. 2016). To investigate the role that chemerin plays, not only in mediating a photoperiodic response, but also as a gateway for a feedback signal from the periphery to the hypothamus, the results in the F344 rat need to be confirmed in non-seasonal animals. To this date, a study in Sprague–Dawley rats has reported that acute injection of chemerin into the arcuate nucleus of the hypothalamus has no effect on food intake and body weight nor the expression of appetite-regulating neuropeptides. This negative result may be explained by the dose of chemerin and the injection site used in this study (Brunetti* et al*. 2011). Later, the same authors used peripheral chemerin injections and showed a reduction in food intake and body weight, but this seemed to have only a minor effect on hypothalamic neuropeptide expression (Brunetti* et al*. 2014).

While these studies showed seemingly contradictory responses due to different routes, mode of delivery and doses used, the results indicate that chemerin might not act directly through known neuroendocrine appetite-regulating pathways to exert its effects. This idea is supported by the finding that mice lacking CMKLR1 show reduced food intake and body weight but no changes in hypothalamic neuropeptides (Ernst* et al*. 2012). An exciting further possibility is that chemerin might play a role in hypothalamic cellular remodelling. Central chemerin administration (acute and chronic) increases expression of vimentin, an intermediate filament protein, which is used as a marker for visualising cells of glial origin such as ependymal cells and tanycytes. Additionally, long-term central chemerin infusion results in morphological changes to the hypothalamus by increasing vimentin immunolabelling of tanycytes (Helfer* et al*. 2016). These results suggest that chemerin plays a pivotal part in hypothalamic remodelling driving long-term changes in body weight and food intake regulation. Together, the studies highlight a critical role for chemerin in the neuroendocrine control of energy metabolism. Further studies are necessary to understand how the hypothalamus integrates chemerin signal and will provide new insights into the physiological basis of appetite regulation.

It is interesting to note that independent of a neuroendocrine role of chemerin, *Cmklr1* mRNA has recently been shown to be upregulated in Alzheimer patients and in mice, it was identified as a receptor for amyloid-β peptides suggesting a potential role of chemerin in the progression of Alzheimer’s disease (Peng* et al*. 2015). Additionally, CMKLR1 expression in the hippocampus and prefrontal cortex of rats was recently linked with depression (Guo* et al*. 2012), possibly through activation by resolvins (Deyama* et al*. 2018) adding yet another level of complexity to the biological role of chemerin. Thus chemerin–CMKLR1 interaction in the brain remains a fascinating avenue for further research.

### Effect on adiposity

**Figure 3:**
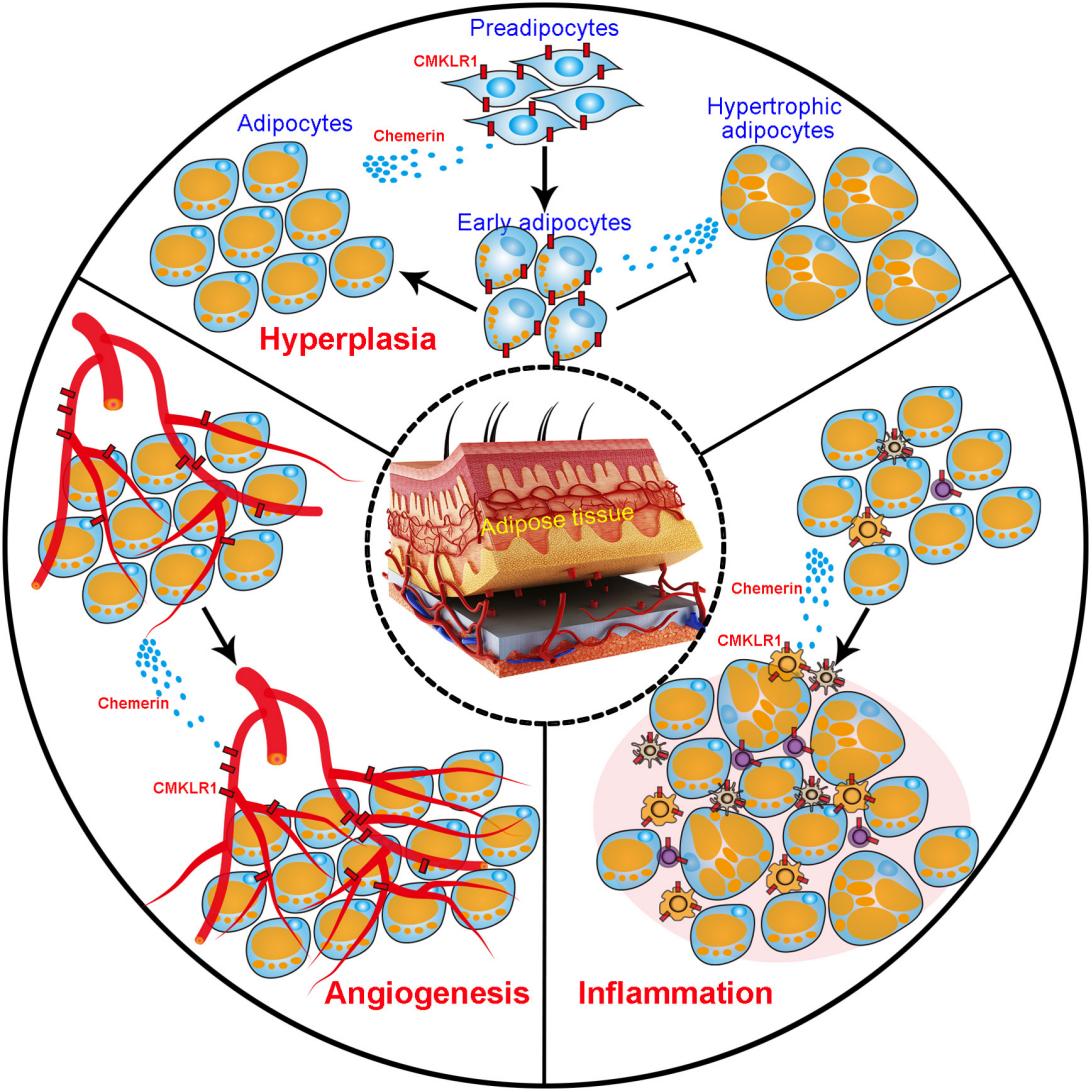
The multidimensional effect of chemerin on adiposity. Multiple roles of chemerin in regulating inflammation, hyperplasia and angiogenesis in white adipose tissue are summarised.

Adiposity is a condition of morbid overweight. Without using body weight, adiposity indexes that include the waist circumference indicate the amount of WAT, a strong predictor of metabolic disorder in humans (Bergman* et al*. 2011). The white fat tissue *per se* releases a plethora of adipokines including chemerin to affect adipose tissue homeostasis, adipocyte metabolism and inflammation in fat tissue (Fig. 3). Chemerin and *Cmklr1* are expressed at high levels in WAT but only in low levels in BAT (Goralski* et al*. 2007). BAT is associated with thermogenesis; hence, this would suggest that chemerin exerts its effect on weight by regulating adipogenesis rather than thermogenesis. However, recently, it has been shown that loss of CMKLR1 suppresses the expression of thermogenesis-related genes in WAT and BAT (Huang* et al*. 2016). Due to its capability to produce heat instead of ATP resulting in weight loss, BAT has been identified as a potential target for treatment of obesity. Retinoic acid is a key player in the regulation of thermogenesis of BAT (Okla* et al*. 2017). Given that chemerin is downstream of retinoic acid signalling, this raises the intriguing possibility that chemerin could promote BAT activity and/or browning of WAT.

#### Targeting adipocytes

Adiposity is featured by an excessive expansion of WAT that relies on an increase in adipocyte size (hypertrophic obesity) and adipocyte number (hyperplastic obesity) (Sun* et al*. 2011). During the differentiation of human preadipocytes into adipocytes, the expression of both chemerin and CMKLR1 is dramatically increased (Roh* et al*. 2007). The activation of chemerin-CMKLR1 axis facilitates the proliferation and differentiation of preadipocytes by inducing the AKT-mTOR and ERK signalling cascade (Jiang* et al*. 2018). Interestingly, chemerin-CMKLR1 signalling, regulated by peroxisome proliferator-activated receptor γ (PPARγ), predisposes the differentiation of bone marrow mesenchymal stem cells (BMSCs) into adipocytes rather than osteoblasts (Muruganandan* et al*. 2011, 2017). Inactivation of chemerin-CMKLR1 signalling by genetic manipulation or neutralisation with antibodies shifts the adipogenic clonal expansion of BMSCs to osteoblastogenic differentiation (Muruganandan* et al*. 2011, 2017). Chemerin treatment also alters the fate of myoblast cells from myogenesis to adipogenesis (Li* et al*. 2015). Consistent with these studies, disruption of *Cmklr1* gene *in vivo* reduces the food intake, body mass and fat deposition of mice (Ernst* et al*. 2012). In contrast, another more recent study showed that *Cmklr1*-knockout mice display mild obesity but normal adipocyte differentiation (Rouger* et al*. 2013). It is found that the number of adipocytes is not changed in the* Cmklr1*-null mice, but there is an increase in the lipid storage in each of adipocytes. Unexpectedly, the differentiation of preadipocytes into adipocytes *in vitro* is not altered by loss of CMKLR1 (Rouger* et al*. 2013). The conflicting data confound our understanding of the role of chemerin in regulating adipogenesis. It is worthwhile to determine whether sex, diet, genetic background and sanitary status of animals influence the body weight and fat deposition of *Cmklr1*-knockout mice, as well as whether the cell type and the culture condition affect the preadipocyte differentiation.

#### Targeting endothelial cells

Adipose tissue consists of adipocytes and vascular endothelial cells, which provide blood supply for the growth of adipocytes (Rupnick* et al*. 2002, Liu & Meydani 2003, Cutchins* et al*. 2012). Remodelling of existing vascular networks and formation of new blood vessels via angiogenesis are required to supply more nutrients and oxygen to the expanding adipose tissue mass (Lijnen 2008). CMKLR1 is expressed in human endothelial cells and is upregulated by proinflammatory cytokines (Kaur* et al*. 2010). Chemerin activates the key angiogenic pathways through PI3K-AKT and MAPK-ERK signalling and induces angiogenesis *in vitro* by promoting the proliferation, differentiation, capillary tube formation and migration of endothelial cells (Kaur* et al*. 2010). The effect of chemerin-mediated activation of MAPK-ERK signalling cascade is dose dependent and the application of MEK1 inhibitor abolishes chemerin-induced angiogenesis (Bozaoglu* et al*. 2010, Kaur* et al*. 2010, Neves* et al*. 2015). The high expression of chemerin has recently been associated with upregulation of vascular endothelial growth factor (VEGF) and tumour angiogenesis (Wang* et al*. 2014, Lin* et al*. 2016). Concomitant with the enhancement of preadipocyte differentiation, chemerin overexpression upregulates VEGF that promotes vascular endothelial cell proliferation, differentiation and angiogenesis in the cultured cells. Moreover, the vascular intensity in the fat pad is increased by the overexpression of chemerin (Jiang* et al*. 2018), suggesting that chemerin boosts angiogenic potential of fat tissues. A genome-wide association study in human population implies that serum chemerin level is highly heritable and significantly associated with single nucleotide polymorphism in *EIDL3* gene, which has been known to regulate angiogenesis (Bozaoglu* et al*. 2010). Thus, the reciprocal interaction between adipocytes and vascular endothelial cells may form a positive feedback loop promoting adipogenesis and obesity.

#### Targeting immune cells

It has been widely accepted that increased adiposity is associated with chronic low-grade systemic inflammation (metainflammation) (Tam* et al*. 2010, Mraz & Haluzik 2014, Catrysse & van Loo 2017). Beyond adipocytes and endothelial cells, adipose tissue contains a number of immune cells (Weisberg* et al*. 2003, DiSpirito & Mathis 2015). CMKLR1 is expressed in numerous immune cells that accumulate in the obese adipose tissue, including plasmacytoid dendritic cells, myeloid dendritic cells, macrophages and natural killer cells (Zabel* et al*. 2005, 2006, Parolini* et al*. 2007). Chemerin was first identified to promote the chemotaxis of immature dendritic cells and macrophages (Wittamer* et al*. 2003, 2005). A recent study confirmed that chemerin recruits circulating dendritic cells into visceral adipose tissue, wherein adipocyte-derived HMGB1 protein activates TLR9 in the dendritic cells and induces the secretion of type I interferons. Subsequently, interferons in turn ignite the proinflammatory response of macrophages (Ghosh* et al*. 2016). The initiation of local and systemic inflammation by the adipocyte–immunocyte crosstalk significantly contributes to insulin resistance and obesity. However, whether and how the inflammation exacerbates adiposity remains unclear. Several studies support a role for macrophages in regulating adipocyte hyperplasia (Keophiphath* et al*. 2009, Zhang* et al*. 2013, Brown* et al*. 2014). One study reported that human preadipocytes exposed to the conditioned medium from activated macrophages exhibited profound remodelling of extracellular matrix (Keophiphath* et al*. 2009). The alteration of extracellular microenvironment significantly increases the proliferation and migration of preadipocytes, which expands the pool of adipocyte progenitors and could increase the adipose tissue in obesity. Another observation is that the macrophages, accumulated around the foci of dying adipocytes, promotes recruitment, proliferation and differentiation of adipocyte progenitors by secreting osteopontin (Lee* et al*. 2013). Macrophages have been shown to form crown-like structure around dying adipocyte after high-fat diet feeding (Kim* et al*. 2014). Further experiments are required to determine whether macrophages drive the high-fat-induced hyperplasia of adipocyte. Together, these studies suggest that chemerin may exacerbate the adiposity by recruiting immune cells to the adipose tissue.

### Effect on glucose metabolism

Type 2 diabetes mellitus (T2DM) is a metabolic disorder featured by insulin resistance ensued by elevated blood glucose (hyperglycaemia). Increased serum chemerin that occurs with obesity is highly correlated with the development of T2DM in humans (Roman* et al*. 2012). Although there is consensus that chemerin regulates glucose homeostasis, its role in regulating glucose tolerance remains unclear owing to the contradictory results derived from various *in vivo* and *in vitro* studies. Given that the insulin signalling pathway is the hub to maintain glucose homeostasis by increasing the uptake of glucose into fat and muscle and reducing the production of glucose in the liver, here, we summarise the function of chemerin in regulating insulin secretion and sensitivity (Table 1).

**Table 1:** Summary of the role of chemerin in glucose metabolism.

Experimental models	Phenotype					Reference
	Glucose level	Fasting insulin	Glucose-stimulated insulin release	Insulin-stimulated glucose uptake	Glucose production	
Chemerin-knockout mice	Normal fasting glucose; impaired glucose tolerance	N.A.	Reduced	Enhanced muscle glucose uptake	Normal basal glucose production, increased clamped glucose production	Takahashi* et al*. (2011)
Chemerin overexpression transgenic mice	Improved glucose tolerance	N.A.	Enhanced	N.A.	N.A.	Takahashi* et al*. (2011)
CMKLR1-knockout mice	Reduced fasting glucose under HFD condition; impaired glucose tolerance	Lower under HFD condition	Reduced	Reduced WAT and muscle glucose uptake	N.A.	Ernst* et al*. (2012)
CMKLR1-knockout mice	Normal fasting glucose and normal glucose tolerance under HFD condition	Normal	N.A.	N.A.	N.A.	Gruben *et al.* (2014)
GPR1-knockout mice	Normal fasting glucose; impaired glucose tolerance under HFD condition	Lower under HFD condition	Reduced	Normal	Increased	Rourke* et al*. (2014)
Chemerin application in mice	Exacerbated glucose intolerance in ob/ob, db/db and HFD mice	N.A.	Reduced in in ob/ob, db/db and HFD mice	Reduced liver glucose uptake in db/db mice	N.A.	Ernst* et al*. (2010)
Chemerin application in 3T3-L1 cells	N.A.	N.A	N.A.	Increased	N.A.	Takahashi* et al*. (2008)
Chemerin application in 3T3-L1 cells	N.A.	N.A	N.A.	Reduced	N.A.	Kralisch* et al*. (2009)
Chemerin application in skeletal muscle cells	N.A.	N.A	N.A.	Reduced	N.A.	Sell* et al*. (2009)

#### Targeting insulin secretion

Both glucose-stimulated insulin secretion from pancreas and insulin-stimulated glucose uptake in peripheral tissues contribute to the proper regulation of glucose tolerance. Chemerin and its receptor CMKLR1 are also expressed in the β cells of the pancreas, implying their role in modulating insulin secretion (Takahashi* et al*. 2011). Indeed, chemerin and CMKLR1-knockout mice display reduced glucose-stimulated insulin release, while the gain-of-function study using chemerin transgenic mice shows enhanced insulin secretion in glucose tolerance tests (Takahashi* et al*. 2011, Ernst* et al*. 2012). Mechanistically, loss of chemerin in mice downregulates the expression of transcription factor MAFA and its downstream target gene GLUT2, which serves as a sensor and transporter for glucose in pancreatic β cells and thereby promotes insulin secretion (Takahashi* et al*. 2011). It is thus speculated that declined expression of GLUT2 in the chemerin-deficient β cells underlies the impaired glucose-stimulated insulin secretion. Interestingly, chronic overexpression of chemerin in low-density lipoprotein receptor (LDLR)-knockout mice does not alter the circulating level of insulin (Becker* et al*. 2010), suggesting the existence of a crosstalk between GLUT2 and LDLR in β cells (Thedrez* et al*. 2016).

The second receptor for chemerin, GPR1, binds chemerin with a similar affinity to CMKLR1 (De Henau* et al*. 2016). The GPR1-deficient mice display not only a mild reduction in glucose-stimulated insulin release but also a significant lower level of fasting serum insulin under high-fat dietary condition (Rourke* et al*. 2014). Although previous studies provide some insight into the role of chemerin–CMKLR1 or chemerin–GPR1 axis in coordinating glucose-induced insulin secretion, the precise mechanisms are still not clear.

#### Targeting insulin resistance

Chemerin has also been reported to regulate insulin sensitivity and glucose uptake (Table 1). It is conflicting that *in vitro* studies using 3T3-L1 adipocytes provide data for both promotive (Takahashi* et al*. 2008) and inhibitory (Kralisch* et al*. 2009) effects of chemerin on glucose uptake. The different dosages, durations of chemerin treatment and culture conditions may cause the discrepancy. In the skeletal muscle cells, chemerin administration inhibits the insulin-stimulated glucose uptake and increases the phosphorylation of insulin receptor substrate 1 (Sell* et al*. 2009). Another *in vivo* study shows that chemerin application exacerbates the glucose intolerance in different mouse models of obesity or diabetes but has no effect on normal lean mice. This study shows that chemerin, upregulated in obesity and diabetes, specifically reduces the glucose uptake by liver but not adipose tissue or skeletal muscle (Ernst* et al*. 2010).

Notably, all of chemerin, CMKLR1 and GPR1 mutant mice display glucose intolerance. Chemerin-knockout mice exhibit impaired insulin sensitivity in adipose tissue and liver, resulting in the elevated hepatic glucose production, reduced glucose uptake by fat and increased blood glucose level (Takahashi* et al*. 2011). Genetic deletion of CMKLR1 in mice leads to reduced glucose uptake by adipose tissue and skeletal muscle but not liver (Ernst* et al*. 2012). Under high-fat diet feeding, loss of CMKLR1 exacerbates the glucose intolerance, increases insulin level and enhances insulin resistance in mice (Huang* et al*. 2016). Consistently, heterozygous and homozygous *Gpr1*-knockout mice fed with high-fat diet develop severe glucose intolerance but exhibit reduced glucose-stimulated insulin level (Rourke* et al*. 2014). Overall, the blood glucose levels are elevated in these mutant mice. However, the underlying mechanisms remain to be explored. In contradiction to these results, another study demonstrated that CMKLR1 deficiency in mice did not change body weight, food intake, serum lipid level or insulin resistance (Gruben* et al*. 2014). Thus, the role of chemerin signalling in glucose intolerance needs to be validated.

#### Targeting hepatic gluconeogenesis

Gluconeogenesis, a metabolic pathway resulting in the generation of glucose from non-carbohydrate carbon substrates, takes place mainly in liver. Despite that the liver weight and histology are not changed in chemerin-null mice, the main regulators of gluconeogenesis including glucose-6-phosphatase, phosphoenolpyruvate carboxylase and transcriptional coactivatior PGC-1α are upregulated by genetic ablation of chemerin (Takahashi* et al*. 2011). As a result, the clamp hepatic glucose production is significantly increased in chemerin-null mice, suggesting that loss of chemerin impairs insulin suppression of hepatic glucose generation.

Further analyses in the *Cmklr1*-knockout mice reveal that loss of chemerin signalling reduces the mRNA expression of proinflammatory cytokines TNFα and IL-1β in the liver, regardless of age or diet (Ernst* et al*. 2012, Gruben* et al*. 2014). Interestingly, CMKLR1 loss alters immune cell infiltration in the livers of mice fed with low-fat but not high-fat diet. Consequently, CMKLR1 deficiency protects against hepatic steatosis in the mice fed with low-fat diet. These results imply that chemerin–CMKLR1 signalling is indeed involved in hepatic inflammation but does not change liver histology. Whether CMKLR1 loss impairs gluconeogenesis requires further study.

## The association of chemerin with metabolic disease in humans

**Figure 4:**
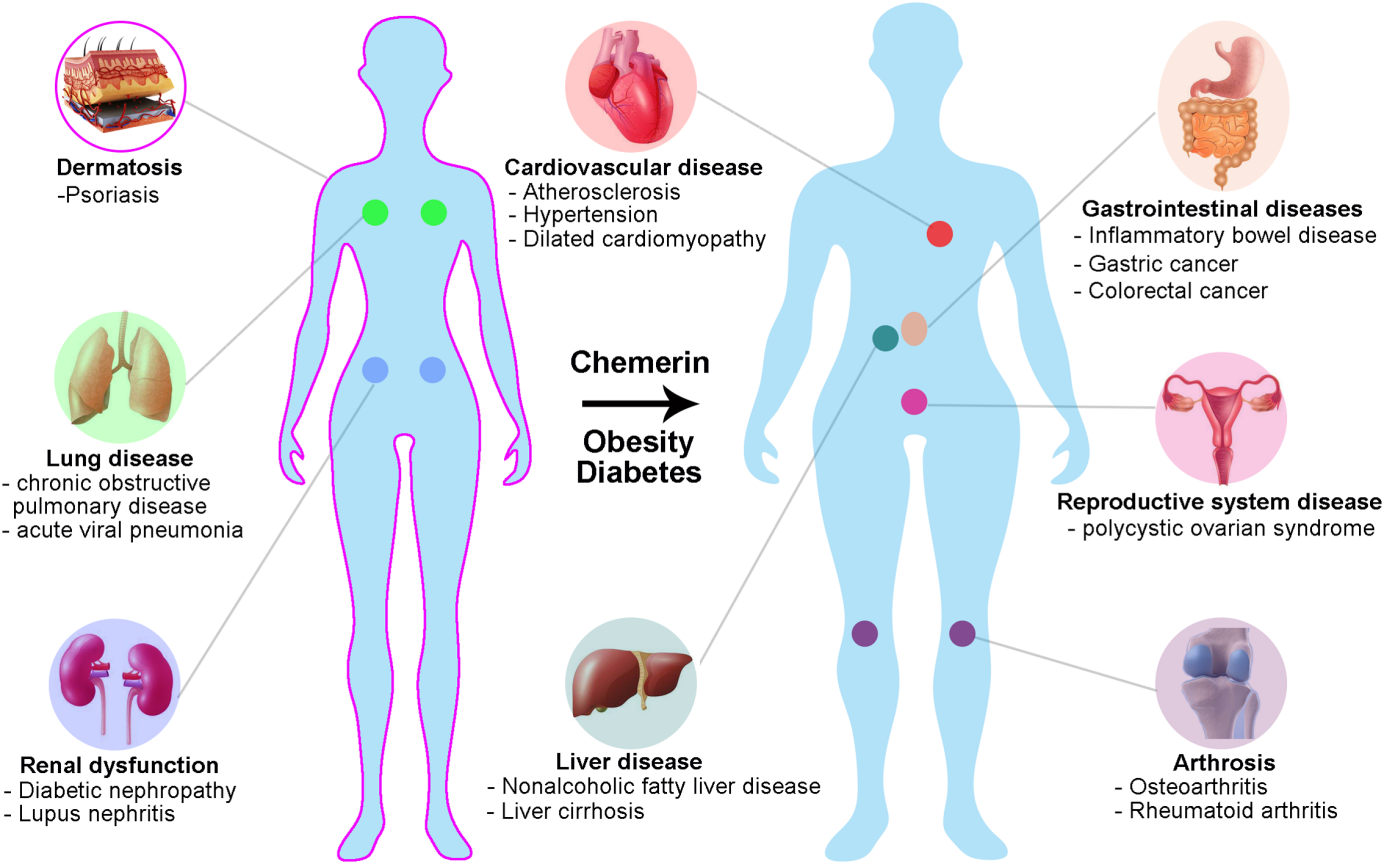
The involvement of chemerin in human diseases. Chemerin is associated with metabolic processes and inflammation and thus its dysregulation plays a critical role in human pathophysiology. Chemerin has hitherto been linked with obesity, diabetes, hypertension, psoriasis, lung disease, renal dysfunction, arthrosis and cardiovascular, gastrointestinal and reproductive disease.

Elevated circulating chemerin is a significant factor for metabolic syndrome. It is hypothesised that chemerin is multidimensionally involved in the pathogenesis of metabolic syndrome by regulating metainflammation, adipocyte plasticity and glucose metabolism in humans. The correlation between chemerin and obesity/diabetes/hypertension seems to be well established. Chemerin has also been associated with many other diseases, including psoriasis, cardiovascular disease and cancer (Fig. 4), reviewed in detail elsewhere (Booth* et al*. 2015, Chiricozzi* et al*. 2016, İnci* et al*. 2016).

### Obesity

A number of human data indicate that systemic chemerin is elevated in obesity. There is a significant and positive correlation between chemerin level and BMI, waist-hip ratio, waist circumference or visceral adipose tissue mass (Bozaoglu* et al*. 2007, Chakaroun* et al*. 2012, Landgraf* et al*. 2012, Shin* et al*. 2012), implying that visceral fat tissue is the primary source for circulating chemerin. A further study shows that the release of chemerin from adipose tissue explants from obese individuals is higher than that in normal-weight controls, and the amount of secretion is linearly correlated with BMI, waist-hip ratio and fat cell volume (Sell* et al*. 2009). Consistent with these studies, systemic chemerin is decreased correspondingly in obese patients who underwent weight loss by diet intervention or bariatric surgery (Sell* et al*. 2010, Chakaroun* et al*. 2012). Interestingly, weight loss by exercise decreases systemic chemerin levels even further, implying that chemerin is a strong predictor for change in insulin resistance in obese adults (Chakaroun* et al*. 2012, Khoo* et al*. 2015, Faramarzi* et al*. 2016). A recent study indicates that exercise alone in elderly, independent of weight loss, has a beneficial effect on chemerin levels (Kim* et al*. 2018).

Although the *CMKLR1* mRNA level in fat tissue is not changed by obesity, the local *RARRES2* mRNA expression in visceral and subcutaneous adipose tissue is significantly increased in obese individuals (Chakaroun* et al*. 2012). It is chemerin expression in omental but not subcutaneous fat tissue that contributes to the elevated systemic chemerin. With the development of ELISAs targeting the four different isoforms of chemerin, it is found that more C-terminal processing of chemerin occurs in adipose tissue of obese patients, resulting in the higher level of bioactive chemerin in local tissue and circulating system (Chang* et al*. 2016). Obese patients are at increased risk of developing numerous comorbid conditions, and increased chemerin levels have been linked to many of these diseases (Fig. 4).

### Diabetes

Recently, novel adipokines such as vaspin, omentin, retinal-binding protein-4, fibroblast growth factor 21, adipocyte fatty acid-binding protein and dipeptidyl peptidase 4 were found to associate with insulin resistance and T2DB in humans (Bergmann & Sypniewska 2013). A collection of clinical studies have also investigated the correlation between chemerin levels and diabetes. While systemic chemerin in patients with T2DM was significantly elevated compared to normal-weight controls in the Caucasian population (Weigert* et al*. 2010), it was not changed or even reduced in Asian T2DM patients without other metabolic complications (Yang* et al*. 2010, Takahashi* et al*. 2013). Importantly, a prospective study shows that the elevation of systemic chemerin precedes the onset of T2DM (Bobbert* et al*. 2015), suggesting that chemerin could serve as a biomarker for early diagnosis of T2DM. Regardless of racial difference, linear regression analyses further reveal a cross-sectional correlation between systemic chemerin in T2DM patients and age, BMI, waist-hip ratio, triglyceride, HOMA-IR, HbA1c, 2-h plasma glucose or blood pressure (Weigert* et al*. 2010, Yang* et al*. 2010, Bobbert* et al*. 2015). The elevation of systemic and local chemerin in adipose tissue is strikingly exacerbated in obese individuals with T2DM (Bozaoglu* et al*. 2009, Sell* et al*. 2010, Chakaroun* et al*. 2012). However, the correlation between circulating chemerin and gestational diabetes mellitus has hitherto remained very controversial (Bozaoglu* et al*. 2009, Hare* et al*. 2014, Guelfi* et al*. 2017, Yang* et al*. 2017). Together, the systemic and local chemerin seems to be upregulated in T2DM patients, especially those with metabolic syndrome.

### Hypertension

Hypertension is recognised as an important constituent of metabolic syndrome. Patients with hypertension have significantly higher levels of serum chemerin (Yang* et al*. 2010, Gu* et al*. 2014). Despite the potential association of chemerin with metabolic characteristics, high chemerin level can serve as an independent predictor of hypertension after adjustment for metabolic risk factors in humans (Gu* et al*. 2014). As abnormal vascular smooth muscle contractility is a major cause of hypertension, chemerin may act on CMKLR1 to mediate vasoconstriction or promote the proliferation of vascular smooth muscle cells (Kunimoto* et al*. 2015, Kennedy* et al*. 2016). A recent study further reveals that chemerin functions through G_i_ proteins to activate L-type Ca^2+^ channel and elicits a dose-dependent calcium influx in vascular smooth muscle cells, which underlies chemerin-induced vasocontraction and hypertension (Ferland* et al*. 2017). A pyridine derivative, Y27632, was found to target a Rho-associated protein kinase (ROCK) and suppress ROCK-mediated Ca^2+^ sensitisation (Uehata* et al*. 1997). Coinciding with the finding that chemerin signals through RhoA/ROCK pathway, ROCK inhibitor Y27632 abolishes chemerin-induced calcium influx and isometric contraction of smooth muscle cells (Rourke* et al*. 2015, Ferland* et al*. 2017). Therefore, upregulation of serum chemerin could result in the vasocontraction via ROCK activation and Ca^2+^ sensitisation in vascular smooth muscle cells.

## Conclusion

Adipokines have recently emerged as potential regulators of appetite and energy homeostasis through endocrine/systemic action in the brain. Circulating levels of adipokines are known to change with increased adiposity and are therefore recognised as contributing factor to the metabolic changes that are seen in obesity and lead to the development of T2DM. One mechanism by which adipokines contribute to T2DM is through inflammation-mediated insulin resistance. As an inflammatory chemokine and adipokine, chemerin has been hypothesised as a link between obesity and the development of T2DM (Ernst & Sinal 2010). Targeting chemerin–CMKLR1 signalling may therefore hold therapeutic potential to improve insulin signalling in T2DM.

### Potential therapeutic role in obesity

Sequence analysis revealed a PPARγ response element within the chemerin (RARRES2) promoter (Muruganandan* et al*. 2011); thus, insulin-sensitising drugs that activate PPARγ might be beneficial for the treatment of obesity and T2DM. In support, in normal and leptin-deficient ob/ob mice, the PPARγ agonist rosiglitazone increased the expression of chemerin transcript in adipose tissue and raised plasma chemerin level (Muruganandan* et al*. 2011, Wargent* et al*. 2015). However, a human study using antidiabetic drugs reported the opposite effect. Pioglitazone and metformin decreased plasma chemerin levels in patients with T2DM (Esteghamati* et al*. 2014). It is therefore not clear whether an agonist or antagonist of chemerin action might be useful for the treatment of T2DM and the contribution of chemerin to the pathology of obesity and T2DM remains elusive.

### Future research

Collectively, these studies illustrate the complexity of chemerin function. Research on this topic is still in its infancy and considerably more work is required to clarify the mechanisms by which chemerin contributes to obesity and associated diseases. Nevertheless, these findings have now opened the field to future studies investigating its precise biological function. Specifically, the pharmacology and signalling properties of chemerin receptors have not been characterised in the hypothalamus. Given the importance of the hypothalamus in sensing and integrating peripheral signals, studies into hypothalamic chemerin signalling might help explain some of the contradictory results that have been obtained. Furthermore, it is important to understand whether chemerin exerts a pro- or anti-inflammatory response or if the effect is indeed bimodal in different biological systems.

In summary, the exciting findings gathered in the last decade clearly highlight an important role for chemerin in the regulation of energy balance and makes it a promising candidate for urgently needed pharmacological treatment strategies for obesity.

## Declaration of interest

The authors declare that there is no conflict of interest that could be perceived as prejudicing the impartiality of this review.

## Funding

This work is supported by funding from the Hundred-Talent Program (Chinese Academy of Sciences), National Natural Science Foundation of China (31771131) and Strategic Priority Research Program of the Chinese Academy of Sciences (XDA16020100) to Q F W.
